# Combustion Synthesis of Materials for Application in Supercapacitors: A Review

**DOI:** 10.3390/nano13233030

**Published:** 2023-11-27

**Authors:** Narek Sisakyan, Gayane Chilingaryan, Aram Manukyan, Alexander S. Mukasyan

**Affiliations:** 1 Institute for Physical Research, National Academy of Sciences of Armenia, Ashtarak-2, Ashtarak 0204, Armenia; sisakyan@gmail.com (N.S.); gayane.chilingaryan.93@gmail.com (G.C.); manukyan.ipr@gmail.com (A.M.); 2 Department of Chemical and Biomolecular Engineering, University of Notre Dame, Notre Dame, IN 46556, USA

**Keywords:** supercapacitors, combustion synthesis, electrochemistry, nanomaterials, composites

## Abstract

A supercapacitor is an energy storage device that has the advantage of rapidly storing and releasing energy compared to traditional batteries. One powerful method for creating a wide range of materials is combustion synthesis, which relies on self-sustained chemical reactions. Specifically, solution combustion synthesis involves mixing reagents at the molecular level in an aqueous solution. This method allows for the fabrication of various nanostructured materials, such as binary and complex oxides, sulfides, and carbon-based nanocomposites, which are commonly used for creating electrodes in supercapacitors. The solution combustion synthesis offers flexibility in tuning the properties of the materials by adjusting the composition of the reactive solution, the type of fuel, and the combustion conditions. The process takes advantage of high temperatures, short processing times, and significant gas release to produce well crystalline nanostructured materials with a large specific surface area. This specific surface area is essential for enhancing the performance of electrodes in supercapacitors. Our review focuses on recent publications in this field, specifically examining the relationship between the microstructure of materials and their electrochemical properties. We discuss the findings and suggest potential improvements in the properties and stability of the fabricated composites based on the results.

## 1. Introduction

The increasing demands for renewable energy have driven the development of novel energy sources, including wind and solar technologies. However, the economic aspects of these approaches are still under consideration partly due to cost and the inability to service during the source period. To make these technologies competitive, research into energy storage systems, such as fuel cells and supercapacitors, has intensified over the last few decades [1,2,3]. The task is to develop an energy storage system that allows cheap energy storage during lean hours and deliver it later.

The supercapacitor, also known as an ultra-capacitor, is an energy storage device that can store and release energy much faster than traditional batteries [4,5,6]. Although they are still inferior to conventional batteries in energy density, they have a higher power density and longer cycle life than conventional batteries. They are widely used in applications that require high power output, such as electric vehicles, renewable energy systems, and consumer electronics [7]. Two main mechanisms of charge accumulation realize the energy storage in supercapacitors:

- non-faradaic, in which the accumulation of charges is realized completely electrostatically via Cologne forces between the electronic charges of the electrode and the ionic charges of the electrolyte at the interface of electrode/electrolyte, thus forming an electrical double layer;
- faradaic where the accumulation of charges is exclusively non-electrostatic in nature and occurs due to the flow of electronic charges resulting from redox reactions between the ions of the electrolyte and the molecules (or atoms) of the electroactive material of the electrode.

Based on these two energy storage mechanisms, the supercapacitors are classified into three types: electric double-layer capacitors (EDLCs) (with non-faradaic or electrostatic charge storage system), pseudocapacitors (with faradaic or electrochemical charge storage system), and hybrid SCs (with the combination of faradaic and non-faradaic charge storage systems).

EDLCs capacitors involve only electrostatic energy storage mechanism (do not involve chemical reactions to store and release energy) thus providing much higher charge/discharge rate than batteries and high power density. The energy density in EDLCs is much higher than in conventional capacitors, which is conditioned with higher specific capacitance (SC) value of EDLCs. As the charge accumulation occurs at the electrode/electrolyte interface, the specific surface area (SSA) of electrode materials plays a crucial role in the SC value of supercapacitors [8].

Different types of carbon-based materials including graphene, activated carbon, mesoporous carbon, carbon aerogel, and carbon nanotubes are used in fabrication of electrodes for EDLCs, as it is well known that such materials provide high SSA and high electrical conductivity, which is another crucial parameter for obtaining high values of SC [9,10,11]. Despite the energy density in EDLCs being much higher than in conventional capacitors, it is still lower than in batteries. One way to enhance SC is to fabricate pseudocapacitors in which the pseudo capacitance arises due to faradaic redox reactions between the electrode active material and electrolyte at their contact interface and can exceed the double-layer capacitance more than 100 times [12,13,14], as electrode materials for pseudocapacitors serve transition metal oxides such as NiO, MnO_2_, Co_3_O_4_, and Fe_3_O_4_ etc. [15,16,17,18]. Although they provide high pseudo capacitance due to redox reactions, they suffer from bad electric conductivity, which obstructs the increase of SC. An effective way to overcome this obstacle and increase the SC is to fabricate hybrid supercapacitors in which hybrid electrodes are developed by integrating the metal oxides with the carbon-based network. In the hybrid electrodes, carbon functions as a conductive channel for charge transfer and improves the overall conductivity, while pseudo capacitance arises from the metal oxides [19,20,21,22,23]. The classification of the supercapacitors is discussed in detail in [3,6,7,19].

Several approaches have been employed to synthesize materials for supercapacitor applications, including hydrothermal/solvothermal, chemical bath deposition, sol-gel, and chemical precipitation processes [24,25,26,27,28,29,30,31,32]. Hydrothermal and solvothermal synthesis methods are similar, except for the solution being aqueous or non-aqueous with much higher temperature need for the latter one and used to synthesize a variety of transition metal oxides, including for pseudocapacitor applications [25,26]. These methods permit fabrication of well crystalline, highly monodispersed particles with controllable size and shape, while typically require high temperature and pressure. Chemical bath deposition is another approach primarily used for preparation nano films and not applicable for all oxides [27,28]. It is low-cost, low-temperature and also suitable for large-scale deposition. Significant efforts are applied to prepare better quality oxide-based nanostructures using the sol-gel process [29,30]. The method is simple, cheap, and allows products of high purity and good homogeneity. Chemical precipitation is also a simple method for large-scale synthesis of nano-size materials including transition metal oxides/hydroxides [31,32]. The challenge is to control the morphology of particles due to the fast precipitation.

Combustion synthesis (CS) is an energy-saving approach that allows easy scale-up and contentious technology for fabricating various materials [33,34,35]. The CS is based on the paradigm of self-sustained exothermic reactions, which take place without any extremal heat sources. Solution combustion synthesis (SCS) is a type of CS method when reagents are mixed on the molecular level as an aqueous solution. SCS is a highly versatile and cost-effective method for fabrication of a wide range of supercapacitor electrode materials. It allows for the synthesis of materials with tailored properties, including particle size distribution, crystallinity, surface area, and chemical composition. By adjusting the composition of the reactive solution, fuel type, and combustion regimes, researchers can fine-tune the properties of the materials produced [36,37,38]. Additionally, SCS generates significant gas release during the synthesis process, which leads to the formation of nanostructured materials with a large specific surface area. This feature enhances the overall performance of the electrode materials. Taking into account its affordability, versatility, and control over material properties, solution combustion synthesis emerges as an attractive method for preparing electrode materials for supercapacitors.

This review critically analyzes the recent publications on the SCS of materials used in construction of SC’s electrodes. We also outline challenges and suggest specific directions of research that will allow the improvement of the electrochemical characteristics of such materials. To better understand the principles of SCS, its driving parameters, and their role in synthesis process, first we will briefly summarize the fundamentals of the method.

## 2. Solution Combustion Synthesis: Fundamentals

**Figure 1:**
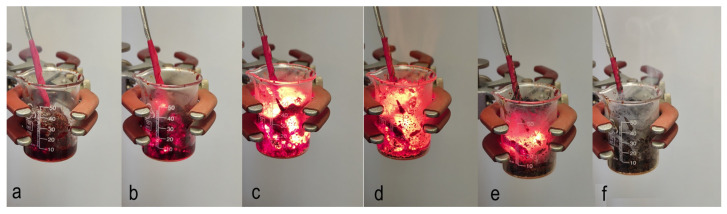
Sequence of frames illustrating the volume combustion synthesis mode: (**a**) initial solution installed slightly above the hot plate; (**b**) preheating stage; (**c**) reaction initiation stage; (**d**) volume combustion synthesis stage; (**e**) cooling stage; (**f**) final product.

The nature of the solution combustion synthesis was described elsewhere [39,40,41]. Initial precursors are solid powders of different oxidizers (typically metal nitrites) and fuels (e.g., glycine, citric acid, and urea). These powders have high solubility in solvent (e.g., water). The reactive solution is the oxidizer and fuel dissolved in a desired ratio in a solvent. The reaction in such a solution can be initiated in two different regimes. Volume combustion synthesis (VCS) implies preheating the whole solution to the self-ignition temperature (T_ig_). After reaching T_ig_, the reaction begins uniformly along the whole volume of the reactive media, resulting in the formation of the desired solid product (Figure 1).

**Figure 2:**
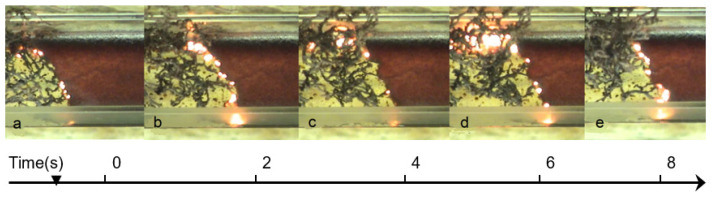
Sequence of frames recorded at different time, (t, s): (**a**) t = 0; (**b**) t = 2; (**c**) t = 4; (**d**) t = 6; (**e**) t = 8; illustrating the self-propagating combustion synthesis mode: reaction propagates from left to right with constant velocity.

The self-propagating high-temperature synthesis (SHS) regime involves local preheating of the solution to initiate the reaction, followed by the combustion wave propagating along the reactive media, leading to the formation of the solid product (Figure 2). In both cases, no external heat sources are required for reaction occurrence after initiation, because a lot of energy is released due to exothermic chemical reactions.

Let us demonstrate the reaction in the system that consists of the aqueous solution of metal nitrite (oxidizer) and glycine (fuel). The overall reaction can be presented in the following scheme:

(1) $${Me}^{\nu }{\left(NO_{3}\right)}_{\nu }\cdot mH_{2}O+2nC_{2}H_{5}NO_{2}+\frac{1}{2}\left(9n-\frac{5}{2}\cdot \nu \right)O_{2}=\;\;\;\;\;\;\;\;\;\;\;\;\;\;\;\;\;\;\;\;\;\;\;\;\;\;\;\;\;\;\;\;\;\;\;\;\;\;\;\;\;\;\;\;\;\;\;\;\;=MeO_{\frac{\nu }{2}}+\left(5n+m\right)H_{2}O+\left(n+\frac{\nu }{2}\right)N_{2}+4nCO_{2}$$

where φ is a valence of the metal; m—number of molecules of tied water in nitrate; 2n—number of moles of fuel used per one mole of oxidizer.

Assigning 18n/5ν = φ, the above equation can be re-written in the following way:

(2) $${Me}^{\nu }{\left(NO_{3}\right)}_{\nu }\cdot mH_{2}O+\frac{5}{9}\varphi \nu C_{2}H_{5}NO_{2}+\frac{5}{4}\nu \left(\varphi -1\right)O_{2}=\;\;\;\;\;\;\;\;\;\;\;\;\;\;\;\;\;\;\;\;\;\;\;\;\;\;\;\;\;\;\;\;\;\;\;\;\;\;\;\;\;\;\;\;\;\;\;\;\;\;\;\;\;\;\;\;\;\;\;\;\;\;\;\;\;\;\;\;\;\;=MeO_{\frac{\nu }{2}}+\left(\frac{25}{18}\varphi \nu +m\right)H_{2}O+\nu \left(\frac{5}{18}\varphi +\frac{1}{2}\right)N_{2}+\frac{10}{9}\varphi \nu CO_{2}$$

It can be seen that if φ equals one, then no external (atmospheric) oxygen is required for complete oxidation of the fuel. For φ < 1 (φ > 1), the fuel-lean (rich) system is considered. For example, for nickel (II) nitrate hexahydrate (m = 6) and glycine system, which is widely discussed in this review, the overall reaction is typically presented as follows:

(3) $$Ni{\left(NO_{3}\right)}_{2}\cdot 6H_{2}O+\frac{10}{9}\varphi C_{2}H_{5}NO_{2}+\frac{5}{2}\left(\varphi -1\right)O_{2}=\;\;\;\;\;\;\;\;\;\;\;\;\;\;\;\;\;\;\;\;\;\;\;\;\;\;\;\;\;\;\;\;\;\;\;\;\;\;\;\;\;\;\;\;\;\;\;\;\;\;\;\;\;\;\;\;\;\;\;\;=NiO+\left(\frac{25}{9}\varphi +6\right)H_{2}O+\left(\frac{5}{9}\varphi +1\right)N_{2}+\frac{2}{9}\varphi CO_{2}$$

The amount of the released heat and thus maximum reaction temperature depends on the chemical composition of the reactive solutions, i.e., parameters φ and m in Equation (2), as well as on parameters of the surrounding atmosphere (pressure, composition: oxygen, nitrogen, air, argon). 

Thermodynamic equilibrium phase composition of the combustion product and corresponding adiabatic (absence of heat losses) combustion temperature may be calculated by using different thermodynamic software packages [42], including the program THERMO, specifically designed for CS systems [43]. To make such calculations, one has to know the standard enthalpies of formation for precursors and all possible combustion products, as well as their specific heats [44].

**Figure 3:**
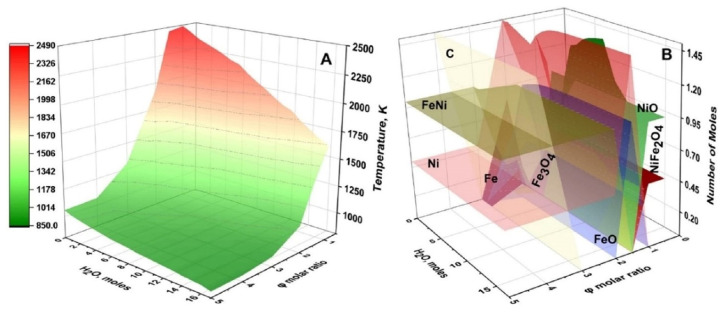
Calculated adiabatic temperature (**A**) and distribution of equilibrium solid products (**B**) for Fe(NO_3_)_3_ + Ni(NO_3_)_2_ + C_6_H_12_N_4_ + H_2_O system depending on C_6_H_12_N_4_/(Fe(NO_3_)_3_ + Ni(NO_3_)_2_) molar ratio (φ) and amount of H_2_O (m). (Reprinted with permission from [45]. Copyright 2023 Springer Nature).

Figure 3 illustrates the specific features of thermodynamic calculations. The φ-m parametric map shows that adiabatic combustion temperature varies between 850–2500 K as a function of the fuel-to-oxidizer ratio and amount of water. It is more important that the phase composition of the equilibrium solid products also change; complex oxide (NiFe_2_O_4_) exists when φ and m are less than one; oxides (FeO, NiO, Fe_3_O_4_) at φ and m are between 1 and 2; and metals/alloys (Ni, Fe, FeNi) are at large values of φ and m.

Volume combustion synthesis is characterized by self-ignition temperature (T_ig_). This temperature is defined by the rates of heat release (q_+_) and heat losses (q_−_) [46]. In the classical case, it is assumed that a homogeneously premixed reactive mixture is placed in a vessel of volume V and the area of the external surface S, that the temperature and concentration are uniform at each point of the vessel, and that heat exchange (q_−_) with the environment occurs according to Newton’s law [47]:

(4) $$q_{-}=-\alpha \left(T-T_{0}\right)S$$

where α is the heat transfer coefficient and T_0_ is the temperature of the environment.

The heat that is generated (q_+_) throughout the volume of the reaction mixture depends on reaction kinetics, which are typically assumed to be of Arrhenius type [47]:

(5) $$q_{+}=k_{0}VQe^{-\frac{E_{a}}{RT}}$$

Q is the heat of the reaction and E_a_ is the activation energy.

**Figure 4:**
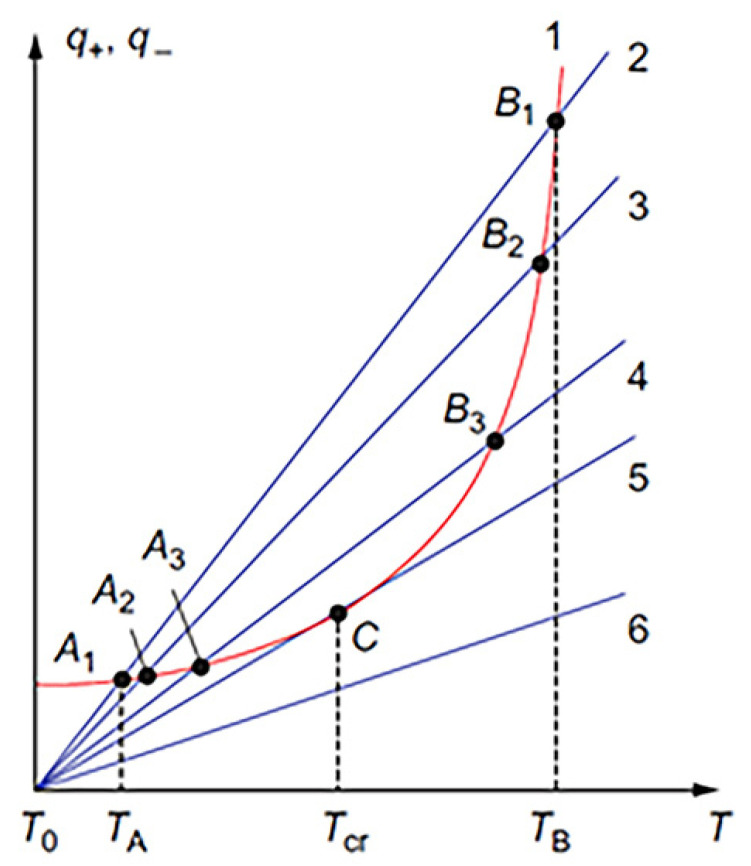
Diagram of thermal explosion in a homogeneous reactive system. (Reprinted with permission from [42]. Copyright 2015 CRC Press Taylor & Francis Group).

Equations (4) and (5) describe the heat balance in the reaction vessel. It is useful to present this relationship graphically (Figure 4). Straight lines 2–6 display the heat losses to the environment with different α, in accordance with Equation (4), and curve 1 shows the rate of heat generation in accordance with Equation (5). Let us consider, for example, conditions 1 and 2, assuming that the initial reaction mixture has the ambient temperature T_0_. Since, at this temperature, the q_+_ is higher than q_−_, the self-preheating process starts and continues until heat balance is reached at point A1, where the rates of heat transfer and heat dissipation are equal:

(6) $$\alpha \left(T-T_{0}\right)S=k_{0}VQe^{-E_{a}/RT}$$

It can be seen that the equilibrium at point A1 is stable. Indeed, in the case of overheating, the heat losses begin to prevail, returning the system to point A1. Similarly, if over-cooling takes place, the heat generation returns the system to point A1. This condition corresponds to the steady-state process in a chemical reactor.

If you reduce the heat transfer coefficient α (lines 3–5), then the point of intersection (A_i_; i = 2,3) shifts to higher temperatures until the heat loss line and heat release curve are tangent at point C. With further decrease of α, the heat release rate (curve 1) is above the heat loss (e.g., line 6) at any temperature. The steady state cannot exist, and the system is self-heated with acceleration. Tangency at point C is the critical condition (T_ig_), which separates the steady-state “slow” reaction from the thermal explosion, i.e., VCS [42]:

(7) $$\alpha S=k_{0}VQ\frac{E_{a}}{RT^{2}}\cdot e^{-\frac{E_{a}}{RT}}$$

Equations (6) and (7) gives equation for critical conditions [42]:

(8) $$T_{ig}=T_{0}+\frac{RT_{ig}^{2}}{E_{a}}$$

The above analysis aims to show that T_ig_ depends on kinetics and boundary conditions. It means that the same reactive solution being under different temperatures of the heating device (e.g., furnace) will have different ignition temperatures. In addition, different reactive solutions typically have different Tig under similar heating conditions. It is also essential that after reaction initiation, the temperature rapidly increases, reaching maximum T_m_ = T_ig_ + Q/cρ (c—specific heat and ρ—density of the products), which is much higher than the temperature of the heating device. The temperature–time history of the system after the reaction (cooling stage) depends on the heat loss conditions. The above conclusions are important because the microstructure and phase composition and thus properties of the synthesized solid products depend on the time–temperature history of the VCS process.

Self-propagating high-temperature (SHS) regime in the homogeneous media (reactive solution) can be described by classical combustion theory [47], which postulates that after local short-term thermal initiation of reactive matter, a combustion wave arises propagating along the media with a certain velocity u. The planar steady-state combustion wave propagation is described by using the equations of thermal conductivity and chemical kinetics [47]:

(9) $$c\rho \frac{\partial T}{\partial t}=\frac{\partial }{\partial x}\left(\lambda \frac{\partial T}{\partial x}\right)+Q\rho \frac{d\eta }{dt}$$

(10) $$\frac{\partial \eta }{\partial t}=k(1-\eta )e^{-E_{a/RT}}$$

where Q, heat of reaction; and η, the degree of conversion.

**Figure 5:**
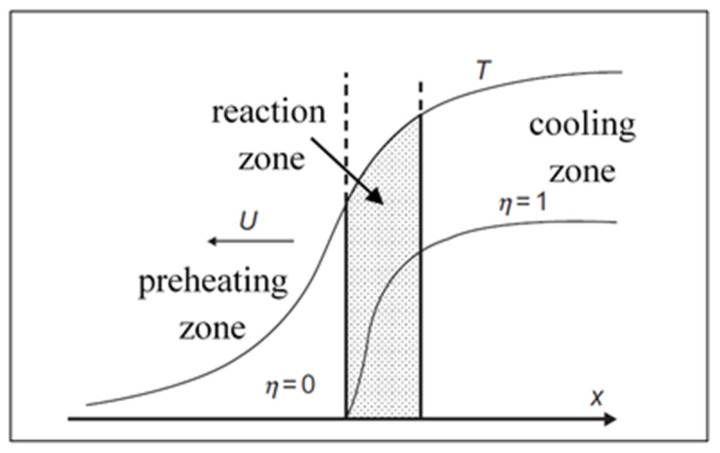
Combustion wave structure: preheating zone (left), reaction zone (shadowed), and cooling zone (right). (Reprinted with permission from [42]. Copyright 2015 CRC Press Taylor & Francis Group).

A typical structure of a combustion wave is given in Figure 5. Due to the strong (exponential) dependence of combustion front velocity on temperature, the chemical transformation happens primarily within the narrow zone in the vicinity of maximal combustion temperature T_m_, which for adiabatic conditions is T_m_ = T_ad_ ~ T_0_ + Q/ρc. In the theory of frontal combustion, a key problem is finding out proper conditions for steady wave propagation at constant velocity u.

Velocity u is an eigenvalue of the problem (9–10) formulated at the following boundary conditions: at X = −∞, T = T_0_, η = 0; X = +∞, T = T_m_, η = 1. Using the relation between the temperature gradient (dT/dx) and the degree of conversion (η) within the reaction zone suggested in [48]:

(11) ηmax−η=λρQudTdx

where λ is thermal conductivity and η_max_ is the degree of conversion at T = T_m_.

Matching solutions for the three zones (Figure 5), one obtains the expression for combustion velocity [48]:

(12) $$u=\sqrt{\frac{\lambda }{\rho Q}\cdot \frac{RT_{m}^{2}}{E_{a}}\cdot e^{-E_{a}/RT_{m}}}$$

The SHS regime is much easier to control. In the steady-state planar combustion wave, the time–temperature history along the whole sample is identical, leading to the uniform microstructure and thus properties of the obtained materials. Both VCS and SHS regimes were used to synthesize a variety of materials, including oxides, nitrides, and alloys [36,49,50]. In this review, we analyze recent reports on SCS of the materials, which have potential applications in supercapacitors. 

## 3. Combustion Synthesis of Materials for SC’s Applications

Many different materials for fabricating electrodes in supercapacitor applications were produced using self-sustained reactions in solutions. Nanostructured transition metal oxides have attracted considerable interest in electrochemistry due to their high theoretical capacity and good chemical stability [15,51,52]. The electrochemical performance of such materials is strongly dependent on their morphology and composition, which significantly influence the transport rate of electrons and structural stability of electrodes in energy conversion and storage systems [53,54,55,56,57]. Transition metal sulfides, as a new class of pseudo-capacitive materials, have also been extensively studied because of their capabilities to provide high energy density. Fabrication of transition metal oxides and sulfides by SCS allows precise control of the microstructure of the fabricated materials [58,59]. Below, we overview recent publications in the SCS field, focusing on the microstructures and electrochemical properties of the fabricated materials. Specifically, we discuss examples of nickel (Ni)- and manganese (Mn)-based compositions, which are considered to be excellent candidates for supercapacitor applications.

### 3.1. Nickel-Based Compositions

Among the materials studied, nickel-oxide is especially attractive because of its high theoretical capacitance, resourcefulness, facile fabrication protocols, low cost, environment friendliness, and good thermal and chemical stability. Many reports have been made on synthesizing various NiO nanostructures, including nanoflowers, nanoflakes, porous nano/microspheres, and nanofibers, prepared via multiple synthesis methods, including SCS (Table 1).

However, pseudo-capacitors based on such materials usually need better rate capability due to the slow faradaic reactions and low electrical conductivity. Despite significant progress in modifying pristine nickel oxide, up to now, there is a great challenge to simultaneously achieve high specific capacitance, rate capability, and cycling stability. One can improve the supercapacitance performance by controlling the material construction, forming composites, and developing new nickel-based materials. Here, we review recent works for the combustion synthesis of Ni-based composites with enhanced electrochemical characteristics.

NiO/ZnO nanocomposite was synthesized by a simple and facile gel combustion method from metal nitrides using citric acid as a fuel, followed by relatively long (3 h) calcination at 500 °C [72]. XRD analysis revealed the presence of cubic NiO and hexagonal ZnO phases, with essentially no impurities. The average crystallite size, estimated by Scherrer’s equation, was ~25 nm. SEM studies showed that the powder has a sphere-like morphology with an average particle size of ~60 nm.

**Figure 6:**
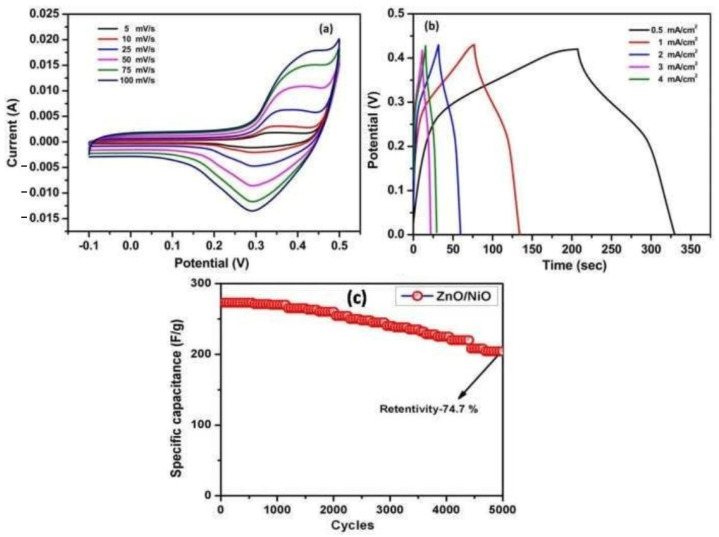
(**a**) Cyclic voltammetry curve; (**b**) charge–discharge curve; (**c**) cyclic stability curve for NiO/ZnO nanocomposites. (Adapted with permission from ref. [72]. Copyright 2020. BEIESP).

Electrochemical measurements were performed in the KOH-based electrolyte by a three-electrode cell setup. Cyclic voltammetry (CV) at the scan rates between 5 mV/s and 100 mV/s displays a typical pseudo-capacitive behavior with characteristic redox peaks (Figure 6a). The calculated values for specific capacitance (SC) were 111, 124, 136, 166, 146, and 198 F g^−1^ for 100, 50, 25, 10, and 5 mVs^−1^ respectively. Figure 6b demonstrates typical Galvano-static charge-discharge (GCD) curves at current densities of 0.5–4 mA/cm^2^. SC values determined by the method were 450, 390, 312, 270, and 174 Fg^−1^ for current densities of 0.5, 1, 2, 3, and 4 mA/cm^2^, respectively. Figure 6c displays the cyclic stability of NiO/ZnO nanocomposites, showing SC retention (SCR) of 74.7% after 5000 cycles and a current density of 3 mA/cm^2^.

The observed effect of an increase of specific capacitance with a decrease in current densities was explained as follows: at lower current densities, ionic charge carriers have enough time to diffuse through the electrode/electrolyte low so fast that the accumulation of ions on the working electrode surface is minimal, resulting in small SC. Thus, the authors demonstrated that a combustion-based approach could fabricate impurity-free complex NiO/ZnO nanopowder (60 nm). The obtained material possesses SC ~400 F/g at current densities 1 mA/cm^2^ and SCR ~75% after 5000 cycles at a current density of 3 mA/cm^2^. 

**Figure 7:**
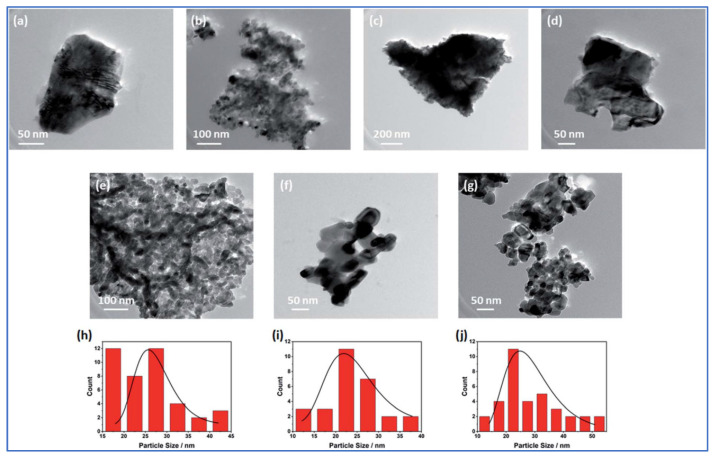
Morphologies of (**a**) NiO; (**b**) Ni_x_Co_1−x_O_y_; (**c**) Ni_x_Fe_1−x_O_y_; (**d**) Ni_x_Mn_1−x_O_y_; (**e**) Ni_x_Mo_1−x_O_y_; (**f**) Ni_x_Cu_1−x_O_y_, and (**g**) Ni_x_Cr_1−x_O_y_. Particle size distribution of (**h**) Ni_x_Co_1−x_O_y_; (**i**) Ni_x_Mo_1−x_O_y_; (**j**) Ni_x_Cr_1−x_O_y_. (Reprinted with permission from [73]. Copyright 2022 The Royal Society of Chemistry).

Nickel-based hybrid metal oxides (Ni_x_Me_1−x_O_y_: Me = Co, Fe, Mn, Mo, Cu, or Cr) were produced by mixing metal nitrates precursors with glycine, followed by VCS mode and calcination at 500 °C for 6 h [73]. The SEM studies show foam-like morphology for all materials. The specific surface area (BET) of all investigated foams was found to be between 6.8 and 83.4 m^2^/g, with pore diameter ranging between 8.5 and 12.3 nm (see Table 2). TEM imaging of the as-prepared oxides reveals that Ni_x_Co_1−x_O_y_, Ni_x_Mo_1−x_O_y_, and Ni_x_Cr_1−x_O_y_ foams involve spherical particles (Figure 7b,e,g, respectively) with an average particle size in the range of 26 to 29 nm. The random agglomerates in flake-like structures were obtained for other oxides (Figure 7a,c,d,f). The most important result of this study is that substituting the Ni in NiO structure with other metals (e.g., by Mo and Cr) may significantly increase the specific surface area of the powder.

In addition, the electrocatalytic performance of the fabricated hybrid Ni-based materials was investigated towards oxygen evolution reaction in an alkaline medium. It was shown that while among all hybrid oxides, the higher BET ~84 m^2^/g was observed for Ni_x_Mo_1−x_O_y_ composition, the Ni_x_Cr_1−x_O_y_ powder with BET ~30 m^2^/g showed the best electrocatalytic performance, exhibiting the lowest (404 mV) overpotential at a current density of 10 mA/cm^2^, onset potential of ~1.6 V, and Tafel slope~53 mV/dec. It means that not only specific surface area but also the synergism between metals is an important parameter to tune up the material’s electrochemical properties.

Complex CNTs/C/NiMoO4 composites were fabricated by one-step volume solution combustion method using citric acid as a fuel and ammonium heptamolybdate (NH_4_)_6_MoO_24_ as molybdenum source [74]. The overall reaction was expected to proceed as follows:

(13) $$7Ni{\left(NO_{3}\right)}_{2}+{\left(NH_{4}\right)}_{6}{Mo}_{7}O_{24}+\varphi C_{6}H_{8}O_{7}+\left(\frac{9}{2}\varphi -13\right)O_{2}\to \;\to 7NiMoO_{4}+6\varphi CO_{2}↑+10N_{2}↑+\left(12\varphi +4\right)H_{2}O$$

To study the effect of the fuel amount on the material’s microstructure and electrochemical properties, the *φ* value in the reactive solution was varied: 6.5/9, 13/9, 26/9, 39/9, and 52/9. The CNTs/C/NiMoO_4_ composites were also obtained by adding an appreciable quantity of acid-treated CNTs (25 mg, 50 mg, and 100 mg denoted as 25-CNTs/C/NiMoO_4_, 50-CNTs/C/NiMoO_4_, and 100-CNTs/C/NiMoO_4_, respectively, into the optimized fuel-to-oxidant ratio 13/9). The influence of φ on the specific surface area, pore volumes, and size of the materials is shown in Table 3.

The thermodynamic calculations suggest that the adiabatic combustion temperature possesses its maximum at the stoichiometric φ value (i.e., 26/9 for the considered system) and decreases with increasing or decreasing of φ value. This effect explains observed changes in the materials’ specific surface area, pore volume, and crystallinity. Well crystalline powder with the lowest BET formed after combustion of the stoichiometric solution (Table 3). It was also shown that in crystalline samples, the observed diffraction peaks correspond to the monoclinic NiMoO_4_, which belongs to a space group of C^2^/m.

**Figure 8:**
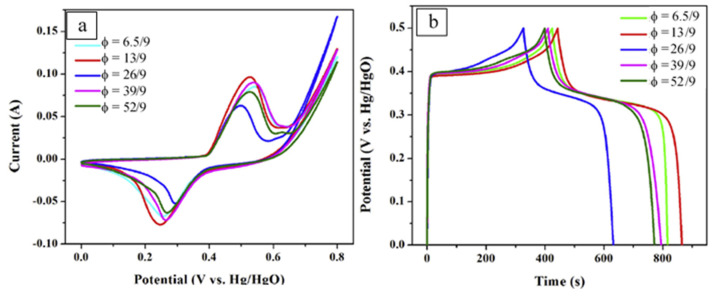
The CV curves (**a**) of samples at scan rate of 10 mV/s and galvanostatic charge–discharge curves (**b**) of samples at the current density of 1 A/g. (Reprinted with permission from [74]. Copyright 2018 Elsevier).

SEM analysis reveals that most samples are porous agglomerates of irregular plate- and grain-like particles. Unlike other materials, the sample obtained from the reactive solution with φ = 13/9 is composed of uniform spherical particles with a narrow size distribution of 20–30 nm. For this reason, this composition was selected to prepare the CNTs/NiMoO4 powders. Electrochemical performance of as-prepared C/NiMoO_4_ composites was studied using CV and GCD in a typical three-electrode system (Figure 8). Analysis of the discharged curves at current density 1 A/g allowed estimation of the specific capacitances of the materials (Table 4).

It can be seen that material fabricated from the reactive solution with φ = 13/9 possesses the highest SC (859 F/g). The addition of the electroconductive CNTs allows an increase in the value up to ~1037 F/g. The asymmetric supercapacitors were fabricated based on the 50-CNTs/C/NiMoO_4_ composites as an anode and the AC as a cathode. The polypropylene membrane and the 2 M KOH aqueous solution were used as the separator and the electrolyte, respectively. Again, the SC was calculated by analysis of the GCD curves. In such a configuration, the CNTs/C/NiMoO_4_//AC exhibited a considerably high SC of ~70 F/g at the scan rate of 0.2 A/g within 0–1.5 V, and the energy density of 32.6 and 18.2 Wh/kg with the power density of 150.5 and 8509 W/kg, respectively. This asymmetric device showed a well-cycle stability, in which the capacitance retained 96.5% after 1500 cycles.

Several outcomes from this work can be outlined:

- The fuel-to-oxidizer ratio is an effective parameter to control the crystallinity and specific surface area of the synthesized materials.
- One may reach sufficient material crystallinity without long-term calcination.
- One may obtain relatively high SC at the specific surface area ~50 m^2^/g.
- The addition of CNTs improves the electrochemical properties.

Various Ni-based carbon-nitrogen containing composites, i.e., Ni_2_(CO_3_)(OH)_2_, Ni_3_(NO_3_)_2_(OH)_4_, NiC_2_O_4_, have been synthesized via a one-step salt (NaCl)-assisted solution combustion synthesis using glucose (C_6_H_12_O_6_) as a fuel at a temperature of 215 °C [75]. The molar ratio of ξ = NaCl: Ni(NO_3_)_2_ was adjusted to be 0, 0.5, 1.0, and 1.5. The XRD patterns of the as-synthesized samples showed that: (i) for a reactive solution with ξ = 0, the main product phase was Ni_3_(NO_3_)_2_(OH)_4_; (ii) for ξ = 0.5, a ternary composite composed of Ni_3_(NO_3_)_2_(OH)_4_, nickel oxalate hydrate NiC_2_O_4_·2H_2_O, and Ni_2_(CO_3_)(OH)_2_·H_2_O formed; (iii) for ξ = 1.0, the NiC_2_O_4_·2H_2_O and Ni_2_(CO_3_)(OH)_2_·H_2_O were the dominant products, with a high amount of the latter phase; (iv) for ξ = 1.0, only the NiO phase was detected.

**Figure 9:**
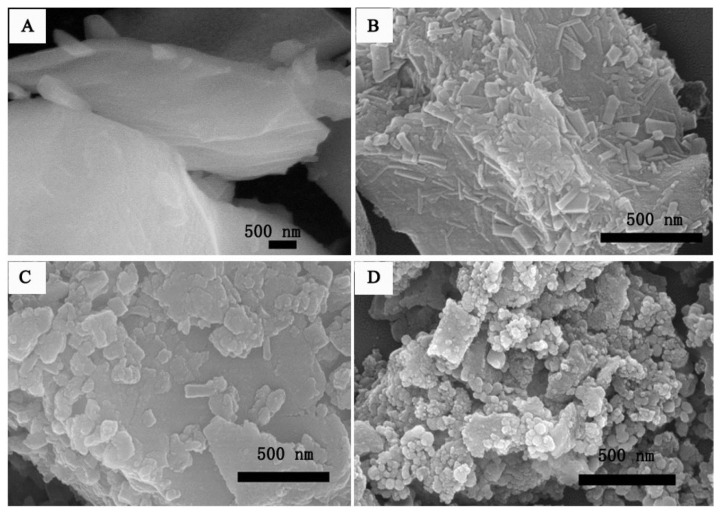
SEM images of (**A**) ξ = 0.0; (**B**) ξ =0.5; (**C**) ξ = 1.0; and (**D**) ξ =1.5. (Reprinted with permission from [75]. Copyright 2018 Elsevier).

SEM images for samples synthesized with various amounts of NaCl show that salt amount affects the morphology of the as-prepared products (Figure 9). Composites fabricated from a solution with ξ = 0.5 (Figure 9B) are composed of rectangular nanoparticles with a length of ~150 nm and a width of ~90 nm. Further increase of the salt concentration (ξ = 1.0; Figure 9C) results in a decrease of a number of rectangular nanoparticles. Combined with the XRD results, these rectangular nanoparticles are identified as NiC_2_O_4_·2H_2_O phase. It was concluded that the formation of NiC_2_O_4_·2H_2_O and Ni_2_(CO_3_)(OH)_2_ phases is related to the presence of NaCl. Thus, the main synthetic outcome of the work is that it was shown that salts (NaCl; NaF; NaBr; NaI; KCl) catalyze the formation of “unusual” Ni-based phases, such as nickel oxalate (NiC_2_O_4_·2H_2_O) and otwayite (Ni_2_(CO_3_)(OH)_2_·H_2_O.

The electrochemical performance of the resulting products was characterized by a standard three-electrode configuration in a 6.0 M KOH aqueous electrolyte. Composite, obtained by salt-assisted solution combustion synthesis from solution with ξ = 1.0, exhibited the highest specific capacitance (1420 F/g at 1 A/g) and cycling performance (60.0% after 2000 cycles) among all synthesized materials (Table 5). A hybrid supercapacitor with a positive electrode made from composite fabricated by using a solution with ξ = 1.0 as a positive electrode and active carbon as a negative electrode delivers high energy density (32.5 Wh/kg at a power density of 310.2 W/kg) and good long-term cycling stability (over 62.5% at 1000 cycles).

Table 5 also summarizes data on SC and CR on different Ni-based materials. It can be concluded that ternary Ni-C-O phases functionalized by high CNTs or graphene are the promising candidates for super capacitor applications.

As mentioned above, nickel sulfide-based composites are other materials to consider for the SC’s application. For example, nickel sulfide/reduced graphene oxide (NiS/RGO) powders were prepared by solution combustion method using nickel nitrate and graphene oxide as oxidants together with thioacetamide (C_2_H_5_NS), as well as glycine as the fuels [81]. It was shown that a 1:1 mole mixture of nickel nitrate and thioacetamide after synthesis resulted in the formation of 100% nickel disulfide (NiS_2_). Adding 2 moles of glycine to this solution led to a composite consisting of 67% nickel sulfide (NS) and 37% NiS_2_. Combustion of reactive nitrate–thioacetamide–glycine solution with a 1:1:3 mole ratio produced 100% of NiS. A NiS/RGO material was fabricated by adding GO to the 1:1:3 mole solution. Graphene oxide (GO) was in situ reduced during the combustion process to produce NiS/RGO nanocomposites. These results again outline the versatility of the combustion synthesis method to one-step synthesis of various composites.

**Figure 10:**
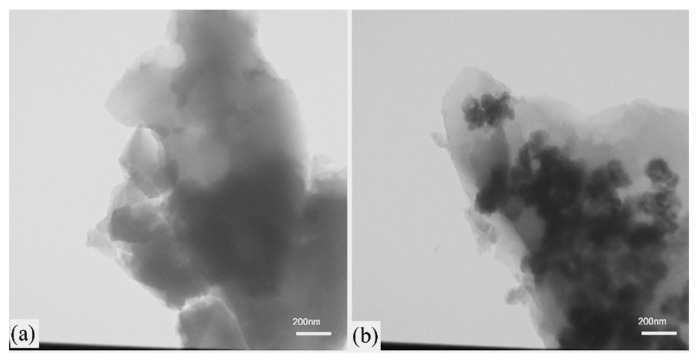
TEM images of the combusted powders: (**a**) NiS and (**b**) NiS/RGO. (Reprinted with permission from [81]. Copyright 2021 Elsevier).

SEM images of nickel disulfide powder show a porous and foamy morphology involving agglomerated spherical nanoparticles (~50 nm). The NiS and NiS/RGO materials possess bulky microstructures formed by strongly agglomerated nanoparticles. TEM images of these materials revealed that NiS nanoparticles (~50 nm) are uniformly distributed on the RGO sheets (Figure 10b).

**Figure 11:**
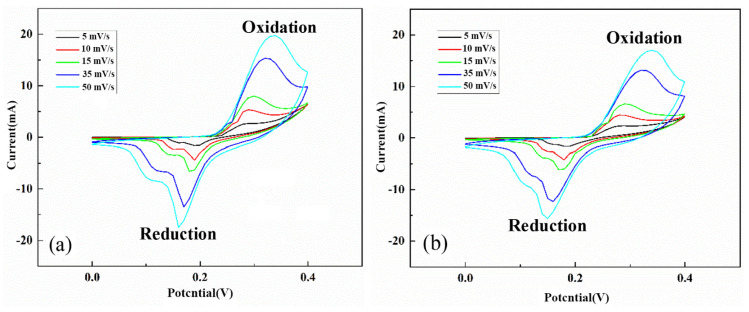
The CV curves of NiS (**a**) and NiS/RGO (**b**) materials at the various scan rates. (Reprinted with permission from [81]. Copyright 2021 Elsevier).

Figure 11 shows the CV curves of the NiS and NiS/RGO materials vs. Ag/AgCl electrode in the potential range of 0 to 0.4 V at the scanning rates of 5 to 50 mV/s. The oxidation and reduction peaks in the CV curves indicate the pseudocapacitive behavior of the samples. The NiS/RGO composite powders showed relatively high SC = 305 F/g at a current density of 1.1 A/g. The high conductivity of RGO and distribution of nickel sulfide nanoparticles on RGO sheets led to the high-capacity retention of 91% after 3000 cycles.

To conclude this section, the fabrication approach based on the self-sustained reactions allows practical synthesis of essentially any Ni-based composites. It was also shown that by varying synthesis parameters, such as fuel-to-oxidizer ratio, type of fuel, and temperature–time schedule, one might control the phase composition, shape, and size of the produced particles. The most critical structural characteristics that influence the electrochemical properties are phase composite, the specific surface area of the powder, and the amount and morphology of the electroconductive part of the composite (e.g., carbon). In Ni-based systems, the SCS permits the synthesis of nanostructured composites with SSA up to 100 m^2^/g and essentially any desired phase composition and amounts of electroconductive phase. The reported recorded values of specific capacitance are up to ~1500 F/g at 1 A/g with capacitance retention in the range of 60–100%, which is dependent on the operational conditions.

#### 3.1.1. Manganese Based Materials

Nanostructured manganese oxide nanoparticles were prepared via the SCS method at 250 °C and 300 °C (denoted as C250 and C300) by using a stoichiometric mixture of manganese acetate dihydrate (C_6_H_9_MnO_6_·2H_2_O) and urea (CH_4_N_2_O) [82]. The material fabricated at a lower temperature (C250) consisted of two phases, i.e., tetragonal hausmannite (Mn_3_O_4_) and magnesium monoxide (MoO). The C300 sample possessed a pure Mn_3_O_4_ structure. The average crystallite sizes for both materials were around 25 nm.

Electrochemical behavior was studied by cyclic voltammetry (CV) in 1.0 M Na_2_SO_4_ aqueous solution at room temperature in a potential window of 0 to 0.5 V (vs. Ag/AgCl) at a scan rate of 100 mV/s. It was shown that the specific capacitance was 128 F/g and 32 F/g for the C250 and C300 samples, respectively. The effect was attributed to the higher specific surface area of the material prepared at lower temperatures. The obtained specific capacitances appeared comparable with those reported for Mn_3_O_4_-based materials fabricated by other methods [83,84].

**Figure 12:**
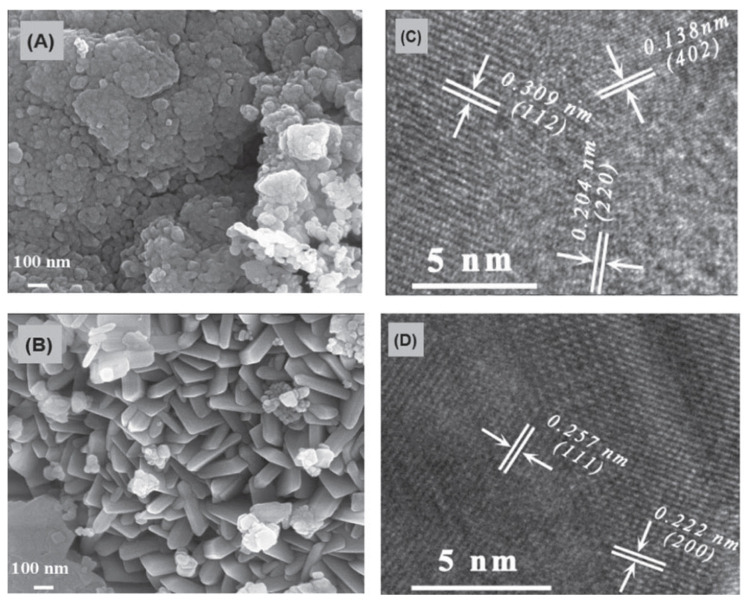
FESEM images of C-Mn_3_O_4_/MnO composites (**A**) and Mn_3_O_4_/MnO composites (**B**); HRTEM images of C-Mn_3_O_4_/MnO composites (**C**,**D**). (Reprinted with permission from [85]. Copyright 2023 IOP Publishing).

The carbon–manganese (C-Mn_3_O_4_/MnO) composites were prepared using a one-step combustion method via initiating the reactions in manganese acetate anhydrous–ethanol solution [84]. In the combustion process, manganese acetate converted into Mn_3_O_4_/MnO (MMs) phases, while the carbon formed by the incomplete combustion of carbonaceous material was embedded into MMs, creating C-Mn_3_O_4_/MnO composites (C-MMs). It was reported that the molar ratio of Mn_3_O_4_ and MnO in C-MMs was 17:3. There were no apparent carbon-related diffraction peaks in C-MMs, which was related to the poor crystallinity of carbon. For comparison, Mn_3_O_4_/MnO (MMs) material was fabricated via the calcination of C_4_H_6_MnO_4_·4H_2_O at 350 °C for 3 h in air. FESEM image shows the spherical-like morphology with an average diameter of about 43 nm for C-MMs (Figure 12a) and platelet structure with a length of around 300 nm for MMs powders (Figure 12b). The HRTEM images of C-MMs (Figure 12c,d) presents the atomic structure. The d-spacing of 0.138, 0.204, and 0.309 nm are assigned to the (402), (220), and (112) planes of Mn_3_O_4_, respectively. The d-spacing of 0.257 nm and 0.222 nm are ascribed to (111) and (200) planes of MnO, respectively.

The electrochemical properties of the as-prepared samples as electrode materials for supercapacitors were evaluated by cyclic voltammetry, charge–discharge, electrochemical impedance, and cycling stability. The calculated specific capacitance of C-MMs was 204 F/g at 1 A/g, which is more than two times larger than that for MMs (90 F/g at 1 A/g). Table 6 reviews the capacitance of manganese oxide-based materials. Charge transfer resistances calculated from Nyquist plots were 0.67 and 42.4 for C-MMs and MMs, which means that C-MMs possess much smaller charge transfer resistance. Charge–discharge cyclic stability for C-MMs at a current density of 1 A/g and within a potential window of −0.1~0.4 V was extremely high (188%) after 5500 cycles.

The influences of fuel type (glycine (G) or urea (U)), fuel to oxidizer ratio (φ), and stabilizer (KCl, KNO_3_) for α-MnO_2_ phase formation via VCS mode were investigated in [92] without any additional calcination steps. When comparing samples prepared using glycine as a fuel with different φ values (φ = 0.15, 0.25, 0.5, 1, 2), the XRD patterns showed Mn_3_O_4_ as the dominant phase for the samples G-1 and G-2 and Mn_2_O_3_ as the dominant phase for the G-0.15, G-0.25, and G-0.5 ones. This effect was attributed to the higher combustion temperature and stronger reduction conditions for a reactive solution with high φ. In the case of urea for φ = 2, the MnO was a predominate phase, while for systems within the φ range 0.8–1, the Mn_2_O_3_ phase dominated. Further decrease of φ leads to the formation of oxides with higher valances, i.e., Mn_2_O_3_ and MnO_2_. At φ = 0.25 and less, the final product involves two phases, i.e., β-MnO_2_ (ICDD 24-0735) and *α*-MnO_2_ (ICDD 44-0141) polymorphs, with a much higher amount of the former phase. The use of KNO_3_ and KCl stabilizers leads to an increase in the amount of *α*-MnO_2_ phase in the final product. For example, adding 2 moles of KNO_3_ to the initial reactants of a U-0.8 sample results in formation a single *α*-MnO_2_ phase. The stability of *α*-MnO_2_ was attributed to the presence of K+ cations.

**Figure 13:**
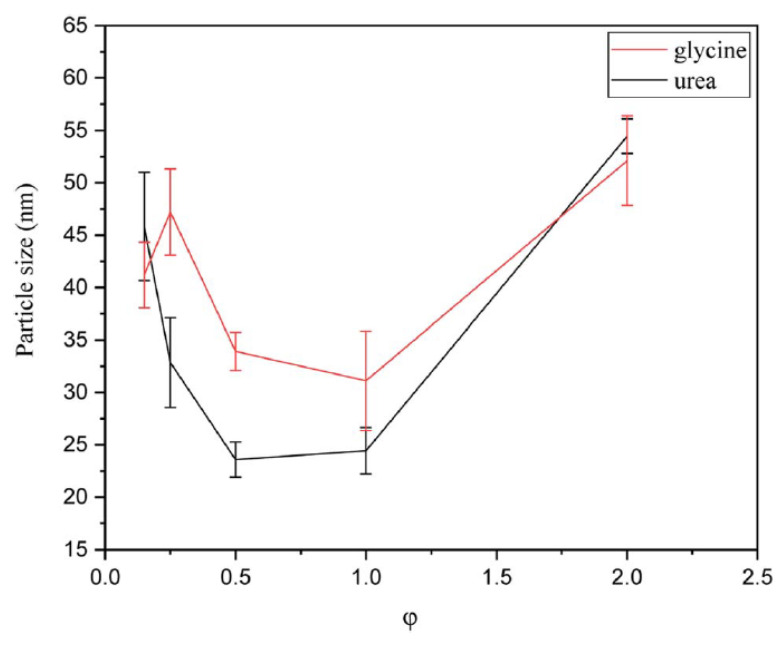
Particle size of the powders synthesized by glycine and urea in various φ ratios. (Reprinted with permission from [92]. Copyright 2023 Royal Society of Chemistry).

**Figure 14:**
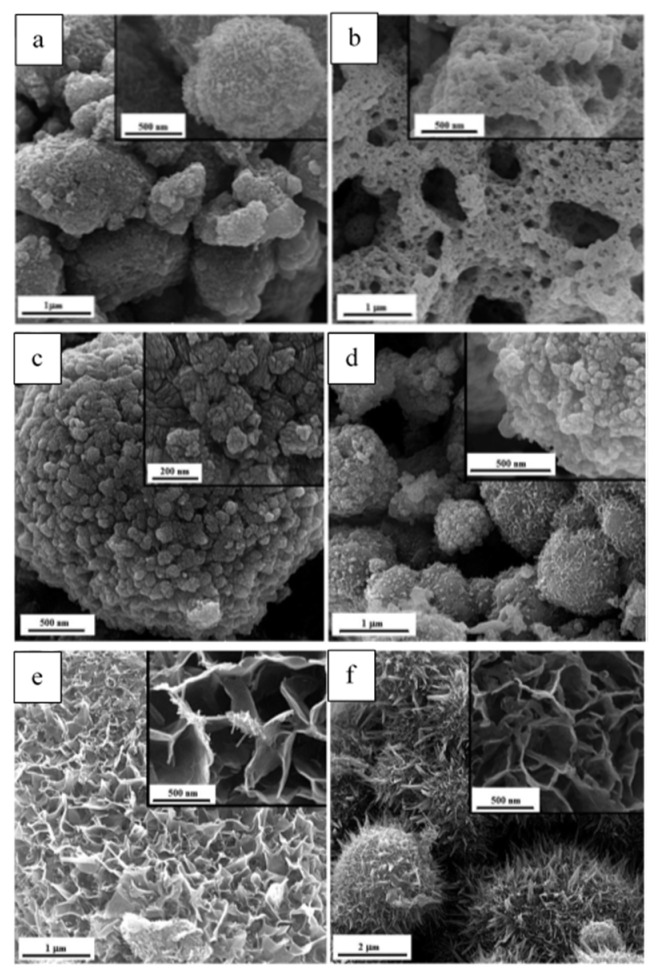
FESEM micrographs of the as-synthesized samples (**a**) G-0.25, and (**b**) G-1; (**c**) U-0.15, (**d**) U-1; (**e**) U-K-0.8, (**f**) U-K-0.9. (Reprinted with permission from [92]. Copyright 2023 Royal Society of Chemistry).

Particle size analysis (Figure 13) shows that the powders synthesized using urea as a fuel had slightly smaller sizes than those fabricated using glycine. This effect was explained by the greater gas volume produced during the synthesis in urea-based systems. According to Figure 14 the smallest particle sizes were obtained for φ = 0.5–1 for urea and φ = 1 for glycine, which correspond to the reactive solutions with the highest synthesis temperature. High temperature led to an increased reaction rate, causing the products to form more rapidly, but also provided faster cooling rates, which may diminish the sintering and agglomeration processes. 

Figure 14 illustrates the morphology of different powders. As shown in Figure 14a, G-0.25 powder reveals a granular morphology, indicating agglomerates of Mn_2_O_3_ nanoparticles with a ridged surface, while the G-1 one (Figure 14b) displays a lacy morphology. The amounts of released gases were higher in sample G-1, which caused greater porosity and more cavities. The FESEM image of the U-0.15 sample (Figure 14c) shows that the MnO_2_ nanoparticles have a cauliflower-like morphology, while U-1 powder (Figure 14d) exhibits a granular morphology. Figure 14e,f illustrates the micrographs of the samples synthesized by adding KNO_3_. A dandelion-like morphology with a needle/flake-like surface was revealed for the α-MnO_2_ nanoparticles. In addition, it was reported that increasing the amount of stabilizer led to a finer morphology of the fabricated materials.

These results demonstrate the versatility of a combustion-based method allowing fabrication of essentially all polymorphs of magnesium oxide with different morphology and particle size. Such studies are an important step to produce materials with defined structure and thus desired properties.

The carbon-coated ZnMn_2_O_4_ nanocrystallites were successfully fabricated by a VSC mode using manganese (II) nitrate tetrahydrate and zinc nitrate hexahydrate (Zn(NO_3_)_2_·6H_2_O) as oxidizers and urea as a fuel. In addition, polyethylene glycol (PEG) was used as a microstructure directing agent [93]. PEG with two molecular weights (200 and 6000) allowed the control of the material pore structure. It served as a precursor for forming a carbon shell on the oxide grains, inhibiting crystallite growth and enhancing the powder’s electronic conductivity. For simplicity, the samples are indexed as ZM-n-Y, where *n* = 2 for PEG-200 used in the synthesis and 6 for PEG-6000, and Y represents the PEG/nitrates weight ratio expressed as Y/12.8. ZM-0 represents the powder synthesized without the addition of PEG.

XRD analysis indicates that all samples exhibit a single tetragonal ZnMn_2_O_4_ phase. The specific surface area (S_total_) and crystallite size (D_c_) are presented in Table 7. The ZM-0 has an average crystallite size of 32 nm, which is larger than for the powders synthesized with PEG addition. The PEG-added powders’ BET-specific surface areas were 27–47 m^2^/g, substantially higher than that (7.8 m^2^/g) of ZM-0. The data indicate that the presence of PEG during synthesis is an effective tool to control powder morphology.

**Figure 15:**
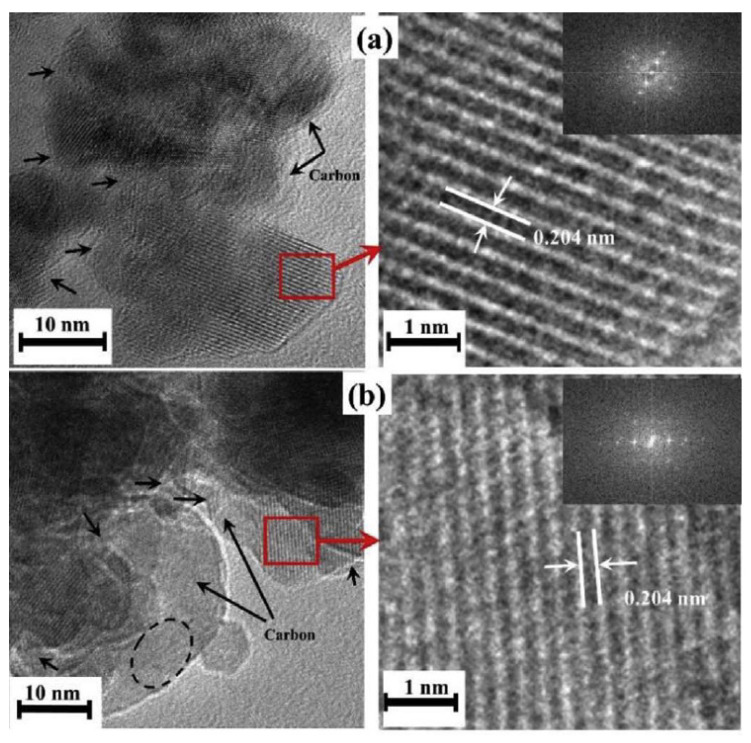
HRTEM images (**left side**) and highly magnified selected regions (**right side**) of (**a**) ZM-6-1; (**b**) ZM-6-2. Inset: FFT patterns of the corresponding regions. (Reprinted with permission from [93]. Copyright 2018 Elsevier).

SEM and TEM studies reveal that the nanoparticles in all samples have an average size of 10–50 nm with nano-porous hierarchical structures. HRTEM images (Figure 15) show the existence of the carbon shell and suggest a high crystallinity of the powders despite a very short synthesis time.

Electrochemical measurements showed that the PEG-added samples had specific capacitances in the 75–150 F/g range, which were substantially higher than that (38 F/g) of the ZM-0 electrode (Table 7). The specific capacitance was found to increase with increasing specific surface area as well as residue carbon content. The ZM-6-1 electrode was mainly used for further characterization. It was shown that the electrode retained a specific capacitance of 69 F/g with an efficiency of 99.4% at a high current density of 5 A/g. A cycle life test of the ZM-6-1 electrode was performed by repeating the GS charge–discharge test between 0.1 and 1.0 V at a current density of 2.5 Ag^−1^. A total of 100% of the capacitance after 5500 cycles was recorded. These results indicate the extremely high electrochemical stability of the Mn-based materials.

A facile large-scale SCS method using glycine as a fuel was reported to produce MnO_2_/MnCo_2_O_4_ composites [94]. The effects of the amount of glycine (φ = 7/9, 14/9, 28/9, 42/9: S1–S4 samples) on the fabricated materials’ phase composition, morphology, and electrochemical properties were investigated. It is reported that all fabricated samples involve fcc spinel MnCo_2_O_4_. The microstructural parameters of the fabricated materials are presented in Table 8.

SEM studies revealed that the morphologies of all samples consist of porous agglomerates of nanoparticles with random granular shapes. The sizes of nanoparticles and agglomerates are 30–50 and 250–300 nm, respectively. All samples have a similar pore size distribution in the 2–4 nm range. The uniform distribution of mesopores should favor electrolyte penetration into the electrode and thus provide more active spots for ion diffusion during the redox reaction.

The S2 powder possessed the smallest nanoparticles (30–35) and agglomerates (200 nm) compared to other samples. This material was selected for the electrochemical characterization. The maximum specific capacitance of 458 F/g is achieved at a current density of 0.5 A/g, with a decent cycling efficiency of 65.5% after 5000 cycles.

**Figure 16:**
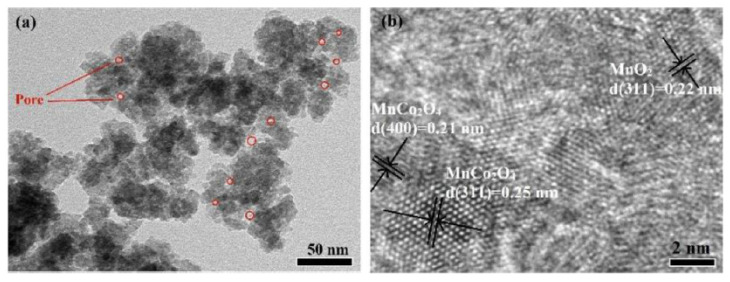
Bright field (**a**) and high-resolution (**b**) TEM images of the MnO_2_/MnCo_2_O_4_ composite. (Reprinted with permission from [94]. Copyright 2016 Elsevier).

To enhance electrochemical performance, manganese–cobalt-oxides with different molar ratios of Mn/Co were also synthesized and investigated on the base of optimum fuel/oxidizer molar ratio. It was shown that by increasing the Mn/Co ratio, the products transformed from spinel MnCo_2_O_4_ granular aggregates to a mixture of MnO_2_/MnCo_2_O_4_ composite. The TEM images of the powder synthesized from the reactive solution with Mn:Co:glycine molar ratio of 3:4:31.5/9 are shown in Figure 16. It was reported that the average particle size was about 28 nm. The maximum specific capacitance of 497 F/g was achieved at a current density of 0.5 A/g and a cycling efficiency of 60% after 5000 cycles. Comparison of supercapacitor performance of the MnCo_2_O_4_-based materials prepared by different methods are summarized in Table 9.

Recall that SCS allows fabrication of essentially all polymorphs of magnesium oxide with different morphology and particle size. Overall, we may conclude that in general manganese-based materials possess less specific capacitance (<1000 F/g) as compared to Ni-based ones (up to 1500 F/g), but they demonstrate excellent SC retaining properties.

#### 3.1.2. Other Compositions

Other nanostructured materials synthesized by the SCS method and tested for electrochemical properties include iron oxide-based and cobalt oxide-based composites. A few recent results are discussed below.

**Figure 17:**
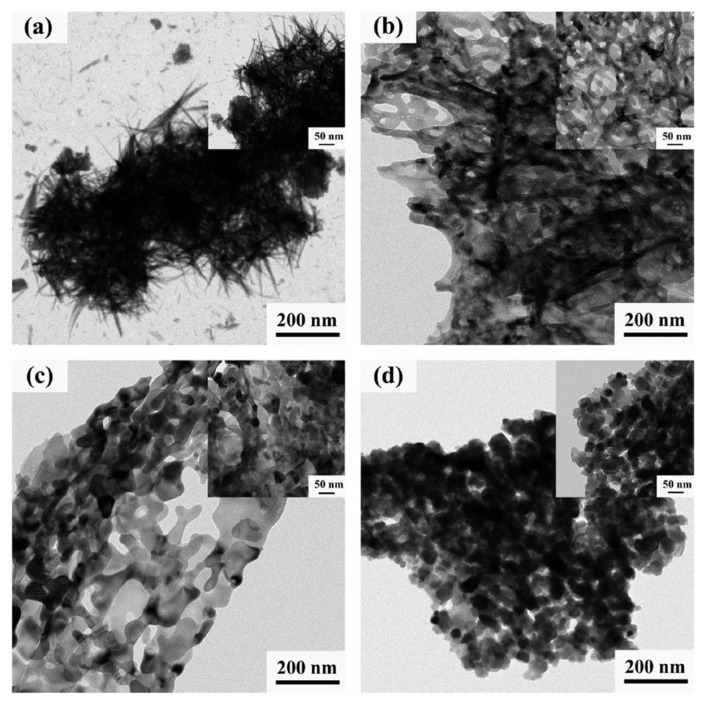
TEM images of SCS products with different φ (the upper-right inset shows the corresponding high magnification image): (**a**) φ = 0; (**b**) φ = 0.5; (**c**) φ = 1.0; (**d**) φ = 1.5. (Reprinted with permission from [106]. Copyright 2018 Elsevier).

The nanostructured iron oxides with controllable morphology and composition were successfully prepared via one-step VCS mode (300 °C) by tuning the fuel (glycine) to oxidizer molar ratio [106]. The TEM images of the fabricated powders are shown in Figure 17. It can be seen that powder obtained by direct decomposition of nitrite (φ = 0) has an urchin-like morphology (Figure 17a). The needle has a large aspect ratio with a diameter of ~5 nm and a length of ~200 nm. The powder produced from the reactive solutions with φ = 0.5 and 1.0 (Figure 17b,c) exhibits a porous sheet-like structure with a large quantity of single ~10 nm pores and a small amount of irregular-shaped pores with a diameter above 50 nm. When the φ value increases to 1.5 (Figure 17d), the produced material involves uniform and well-dispersed nanoparticles of nearly hexagonal geometry with an average diameter of ~50 nm. The specific surface area and pore size of the products were:

- 27 m^2^/g and wide pore size distribution with a maximum at 20 nm (φ = 0).
- 54 m^2^/g and narrow pore size distribution with peak at ~3 nm (φ = 0.5).
- 56 m^2^/g and pore size 2 nm (φ = 1.0)
- 18 m^2^/g and the main peak of pores diameter at ~5 nm (φ = 1.5).

XRD and XPS analysis reveal that the phase composition of the products changed from amorphous α-Fe_2_O_3_ for φ = 0 case to crystalline α-Fe_2_O_3_ for φ = 0.5 and α-Fe_2_O_3_/Fe_3_O_4_ composites for larger φ, with a graduate increase of the amount of Fe_3_O_4_ with a rise in the amount of fuel.

**Figure 18:**
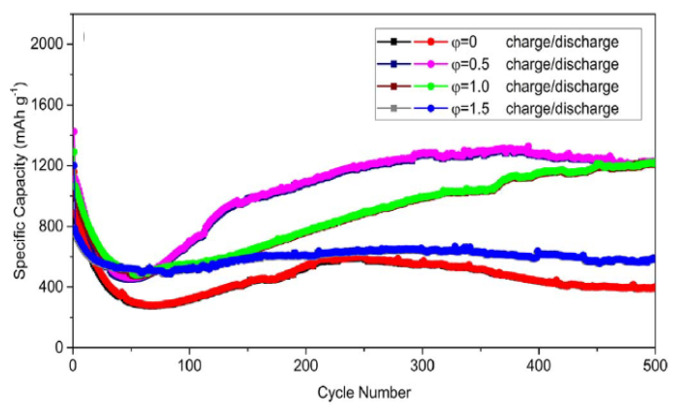
Cycling performance of different φ anodes at the current density of 1 A/g. (Reprinted with permission from [106]. Copyright 2018 Elsevier).

Figure 18 shows the cycling performance of different anodes at the current density of 1 A/g. As can be seen, the φ = 0.5 and 1.0 anodes exhibit a sharp decline in discharge specific capacity in the initial 50 cycles, and then the SC increases significantly to ~1200 mAh/g after 500 cycles. In contrast, for the φ = 0 and 1.5 anodes, a slight decrease in discharge capacity can be detected in the initial 50 cycles. Then, the SC stabilizes at ~400 mAh/g and ~600 mAh/g, respectively. The high SC and superior capacity recovery in φ = 0.5 and 1.0 anodes were ascribed to the porous nanosheets morphology and high specific surface area. The porous nanosheets can shorten the transport length of the Li+ band and also supply favorable accessibility for electrons. In turn, the high specific surface area can provide an appropriate electrode/electrolyte interface to facilitate fast charge transfer and minimize polarization effects. For comparison, Table 10 shows the electrochemical performance of different iron oxide anodes reported in the open literature [14,40,41,42,46,47,48].

**Figure 19:**
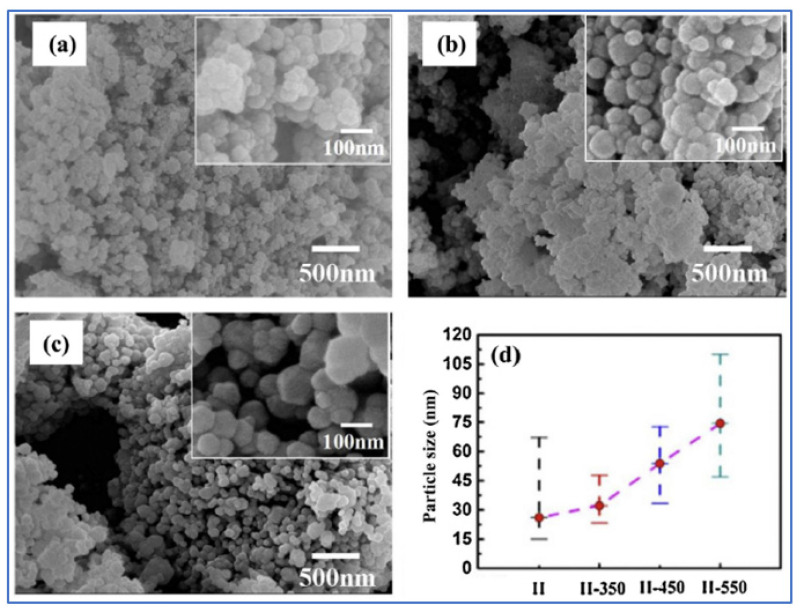
SEM images of samples II-350 (**a**); -II-450 (**b**) and -II-550 (**c**). Part (**d**) shows the average particle size and particle size range. (Reprinted with permission from [114]. Copyright 2014 Elsevier).

Co_3_O_4_ and Co_3_O_4_/CoO nanoparticles have been synthesized by a one-step VCS mode [114]. The molar ratios of citric acid monohydrate and Co(NO_3_)_2_·6H_2_O were fixed to be 3.5/27, 7/27, 14/27(stoichiometry), 21/27 (samples I–IV, correspondingly). The ammonium nitrate (NH_4_NO_3_) was used as an additional oxidizer to support the combustion reaction. Some microstructural parameters of the fabricated powders are shown in Table 11. It can be seen that the maximum SSA ~21 m^2^/g was obtained for sample II (φ = 7/27). SEM images of as-synthesized sample II and after annealing at different temperatures (350; 450; 550 for 3 h) are shown in Figure 19.

The CV studies of sample-II before and after annealing at a scan rate of 5 mV/s revealed that all materials possess pseudo-capacitance features with distinct anodic and cathodic peaks. In addition, the sample-II-350 displays a much higher redox peak current density and larger enclosed area than the others, implying better capacitor performance. The reported SC values for sample-II, -II-350, -II-450, and -II-550 were 155, 391, 108, and 65 F/g, respectively. The analysis of the GSC curves obtained at a constant discharge current density of 0.2 A/g gave the following SC values: 180, 363, 121, and 63 F·g^−1^, for sample-II, -II-350, -II-450, and -II-550, respectively. Sample-350 also demonstrated decent rate performance (286 F/g at 4 A/g) and cycling stability (73.5% retention after 1000 cycles). The results suggest that the electrochemical performance of the material was significantly improved by its annealing at 350 °C for 3 h under a nitrogen atmosphere. The further increase of annealing temperatures resulted in rapid deterioration of the electrochemical performance.

The above examples demonstrate that FeO and CoO-based nanocomposites can be effectively synthesized by the SCS method. Adding ammonium nitrate is a powerful approach to control combustion, which can be used in any SCS system. Optimization of the calcination stage should also be considered, while we believe that materials with desired microstructure and properties can be produced in one SCS step without additional thermal treatment.

## 4. Future Prospects

The solution combustion synthesis involves multiple parameters that can be utilized to control these structural characteristics. However, researchers have only explored a few of them. For instance, all the materials discussed in the review were synthesized using the volume combustion synthesis mode. This mode involves preheating the reactive mixture to a self-ignition temperature, followed by a thermal explosion of the media. Although this mode is easy to accomplish, it is not the optimal choice for controlling the product’s microstructure. Firstly, it is impossible to uniformly preheat the entire volume, leading to non-uniform synthesis conditions. Secondly, the immediate gas release from the large volume results in an uncontrolled dispersion of the media, again resulting in non-uniform conditions.

On the other hand, the steady-state self-propagating mode (SHS) is a much more controllable approach for material synthesis. The temperature–time schedule remains constant throughout the process, as the reaction front moves progressively. The combustion theory allows for precise prediction and variation of the conditions. Therefore, the first recommendation is to utilize the SHS mode to produce desired materials.

An analysis of the existing literature suggests that the main parameter used by researchers to alter the structural characteristics of materials is the fuel-to-oxidizer ratio (φ). While this is indeed an important parameter, there are several others that can be used to control the process:

i. Amount of bound water in the reactive system, which can affect the phase composition of the materials.
ii. Preliminary drying of the reactive media, which may lead to the formation of specific organic complexes and allow for the fabrication of metastable phases that are challenging to produce using conventional methods.
iii. Oxygen-free fuels, which enable the production of not only oxides but also other types of materials such as metals, alloys, and nitrides.
iv. Gas pressure in the reactor, which can influence the kinetics of combustion reactions during SCS and affect the particle size of the resulting materials.
v. Gasifying agents that can increase the specific surface area of the powder.
vi. Different types of solvents that can serve as oxidizers or fuels, intensifying or inhibiting the process.
vii. Impregnation of the reactive solution into high surface area conductive media (e.g., carbon), followed by SHS, resulting in the one-step formation of hybrid MeOx-C structures suitable for electrochemical applications.
viii. Different types of fuels were used in SCS reactions including glycine (CH_4_N_2_O), citric Acid (C_6_H_8_O_7_), urea (CH_4_N_2_O), sucrose (C_12_H_22_O_11_), Glycose D-(+)-C_6_H_12_O_6,_ hydrazine (H_2_N-NH_2_), carbohydrazide (CH_6_N_4_O), oxalyhydrazide (C_2_H_6_N_4_O_2_), and metal hydrazinecarboxylates hydrates (see Table 1 in [115]). The fuel influences the combustion temperature and, hence, the chemical reaction rate and the chemical environment, including the amount of released gas and oxygen concentration. Higher combustion temperatures can lead to higher crystallinity, reduced impurities, and improved structural properties of the resulting materials. However, excessively high temperatures can also lead to sintering or agglomeration of nanoparticles, which may negatively affect the specific surface area. Faster combustion rates and larger gasification typically lead to smaller particle sizes and finer pore structures, which enhance the surface area of synthesized materials, making them more suitable for supercapacitor applications. Using different fuels can optimize the synthesis process and obtain materials with the desired characteristics, such as high surface area, good electrical conductivity, and suitable electrochemical performance for supercapacitor electrodes.

Furthermore, optimizing the SCS conditions allows for the fabrication of materials with desired properties directly in the combustion wave, without the need for any post-thermal treatment. The materials produced are typically well crystalline due to the high temperature in the combustion wave. However, the duration of the high-temperature stage is short, resulting in small particle size powders. Additionally, by controlling the amount of gas phase released during combustion wave propagation, it is possible to obtain materials with high surface area.

The researchers in the field of electrochemistry should take advantage of the extensive knowledge accumulated over more than 50 years of combustion synthesis. By utilizing this knowledge, it is possible to fabricate materials with exceptionally high electrochemical properties.

## 5. Conclusion Remarks

The synthetic approach, which relies on self-sustained chemical reactions, is a powerful method for creating a wide range of nanostructured materials that are applicable in electrochemistry. There are several advantageous aspects to the combustion-based methods to fabricate electrodes for supercapacitors:

- Versatility: This technique allows for the synthesis of materials with any phase composition.
- Simplicity and energy savings: Complex materials can be produced in a single step with minimal external energy usage.
- Easy scaling up and continuous production: The process can be easily expanded and implemented on a larger scale.

Based on an overview of the topic, the following structural characteristics are crucial for electrochemical applications:

- Phase composition, which takes into account the synergy between different elements.
- Specific surface area.
- Particle and pore size distribution.
- Morphological interaction between oxides and electroconductive phases, such as various carbonaceous materials.

All the above parameters can be precisely controlled by using fundamental knowledge on the combustion phenomenon. Thus, this review suggests that SCS is an attractive approach towards fabrication of materials for supercapacitor applications.

## Figures and Tables

**Table 1:** NiO prepared using various methods and their supercapacitor performance. (Reprinted with permission from [52]. Copyright 2016 Elsevier B.V. All rights reserved).

Synthesis Technique	Morphology	SC	SSA (m^2^/g)	Ref.
Hydrothermal	Nanoparticles	609.5 F/g at 5 A/g	58.5	[60]
Hydrothermal	Nanowires	348 F/g at 10 mV/s	85.18	[61]
Hydrothermal	Nanoflakes	137.7 F/g at 0.2 A/g	107.5	[62]
Hydrothermal	Nanocolumns	390 F/g at 5 A/g	102.4	[63]
Sol-gel	Nanoflowers	480 F/g at 0.5 A/g	130	[29]
Sol-gel	Xerogels	696 F/g at 2 mA/cm^2^	-	[64]
Chemical bath deposition	Porous hollow sphere arrays	311 at 1 A/g	325	[65]
Chemical precipitation	Ball-shaped mesoporous structures	124 F/g at 0.1 A/g	477.7	[32]
Chemical precipitation	Hollow spheres assembled from nanosheets	415 F/g at 3 A/g	62	[66]
Electrochemical deposition	Nanoplatelets	112 F/g at 10 mV/s	N/A	[67]
Electrochemical deposition	Nanoflakes	351 F/g at 10 mV/s		[68]
Microwave	Flower-like hollow nanospheres	585 F/g at 5 A/g	176	[69]
Microwave	Hierarchical porous ball-like surface	420 F/g at 0.5 A/g	125	[70]
SCS	Spherical grains	295 F/g at 1 mV/s	-	[71]

**Table 2:** Textural properties of NiO and the hybrid binary Ni_x_M_1−x_O_y_ materials. (Reprinted with permission from [73]. Copyright 2022 The Royal Society of Chemistry).

Catalyst	Surface Area (m^2^ g^−1^)	Pore Volume (cm^3^ g^−1^)	Pore Diameter (nm)
NiO	17.3	0.098298	11.4
Ni_x_Co_1−x_O_y_	9.8	0.043892	9.0
Ni_x_Fe_1−x_O_y_	8.4	0.037339	8.9
Ni_x_Mn_1−x_O_y_	6.8	0.035503	10.5
Ni_x_Mo_1−x_O_y_	83.4	0.354888	8.5
Ni_x_Cu_1−x_O_y_	7.4	0.043915	11.9
Ni_x_Cr_1−x_O_y_	29.8	0.183563	12.3

**Table 3:** Microstructure parameters of the samples. (Reprinted with permission from [74]. Copyright 2018 Elsevier).

φ—CNTs (mg)	BET, m^2^/g	Pore Volume, cm^3^/g	Average Pore Size, nm	Crystallinity
6.5/9—0	59	0.106	3.78	amorphous
13/9—0	48	0.104	3.82	poor crystalline
26/9—0	25	0.064	3.80	well crystalline
39/9—0	46	0.095	4.32	poor crystalline
52/9—0	47	0.130	3.79	amorphous
13/9—50	45	0.110	12.01	poor crystalline

**Table 4:** Some structural and electrochemical characteristics. (Reprinted with permission from [74]. Copyright 2018 Elsevier).

φ—CNTs (mg)	BET, m^2^/g	Crystallinity	Specific Capacitances, F/g
6.5/9—0	59	amorphous	798
13/9—0	48	poor crystalline	859
26/9—0	25	well crystalline	617
39/9—0	46	poor crystalline	780
52/9—0	47	amorphous	758
13/9—50	45	poor crystalline	1037

**Table 5:** Capacitive performance of different Ni-based electrode materials. (Reprinted with permission from [75]. Copyright 2018 Elsevier).

Material	Specific Capacitance, Fg^−1^	Capacitance Retention, %	Ref.
ξ = 0; Ni_3_(NO_3_)_2_(OH)_4_	1260 at 1 A/g	53.6 after 2000 cycles at 10 A/g	[75]
ξ = 0.5; Ni_3_(NO_3_)_2_(OH)_4_ NiC_2_O_4_·2H_2_O, Ni_2_(CO_3_)(OH)_2_·H_2_O	1166	N/A	[75]
ξ = 1.0; NiC_2_O_4_·2H_2_O, Ni_2_(CO_3_)(OH)_2_·H_2_O	1420	60 after 2000 cycles at 10 A/g	[75]
ξ = 1.5; NiO	536	N/A	[75]
Ni_2_(CO_3_)(OH)_2_ microspheres	1178 at 0.5 A/g	108 after 1000 cycles at 80 mV/s	[76]
Ni_2_CO_3_(OH)_2_/functionalized graphene	1508 at 1 A/g	100 after 3000 cycles at 10 A/g	[77]
Ni_3_(NO_3_)_2_(OH)_4_	1202 at 50 mA/g	68 after 1000 cycles at 5 mA/g	[78]
Ni_3_(NO_3_)_2_(OH)_4_/ZnO	1310 at 15.7 A/g	84 after 5000 cycles at 23.6 A/g	[79]
NiO/mesoporous carbon nanospheres	406 at 1 A/g	91 after 10,000 cycles at 3 A/g	[80]

**Table 6:** Comparison of properties of manganese oxide-base electrodes. (Data taken from the references cited in the last column of the table).

MnO_x_ Material	Electrolyte	Rate	SC (F/g)	Cycles	Ref.
Graphene/MnO_2_	1.0 M Na_2_SO_4_	1.0 A g^−1^	205	2000	[86]
Mn_2_O_3_ particle	6.0 M KOH	0.5 A/g	70	1000	[87]
MnO/Mn_2_O_3_	1.0 M Na_2_SO_4_	1.0 A/g	113	500	[88]
Mn_3_O_4_/Mn_2_O_3_	1.0 M Na_2_SO_4_	1.0 A/g	150	500	[89]
Mn_3_O_4_/graphene	1.0 M Na_2_SO_4_	1.0 A/g	130	500	[90]
Mn_3_O_4_/graphene quantum dots	6.0 M KOH	2.0 A/g	182	100	[91]
C-Mn_3_O_4_/MnO	6.0 M KOH	1.0 A/g	204	3200	[85]

**Table 7:** Structural characteristics specific capacitance of C-ZnMn_2_O_4_ materials. (Reprinted with permission from [93]. Copyright 2018 Elsevier).

Powder	S_total_, m^2^/g	D_c_, Nm	Pore Volume, cm^3^/g	Carbon, wt.%	SC, F/g
ZM-0	7.8	32	0.05	0	38
ZM-2-1	47	8	0.08	2.78	130
ZM-2-5	44	11	0.1	1.29	92
ZM-6-1	42	10.4	0.07	2.04	150
ZM-6-2	46	10.8	0.09	1.83	137
ZM-6-5	27	20.8	0.1	0.05	76

**Table 8:** Microstructure parameters obtained by BET analyses of the samples. (Reprinted with permission from [94]. Copyright 2016 Elsevier).

Sample	BET (m^2^/g)	Average Pore Size (nm)	Total Pore Volume (cm^3^/g)	Micropore Area (m^2^/g)
S1	99.30	3.40	0.166	8.76
S2	88.53	3.81	0.113	6.66
S3	25.19	3.82	0.062	2.10
S4	11.33	3.80	0.033	1.76

**Table 9:** Specific capacitance of MnCo_2_O_4_-based materials prepared by different methods. (Reprinted with permission from [94]. Copyright 2016 Elsevier).

Material	Prepare Method	Capacity, F/g	Ref.
MnCo_2_O_4_._5_@δ-MnO_2_ hierarchical nanostructures	HTM	357.5 (0.5 A/g)	[95]
MnCo_2_O_4_@reduced graphene oxide	HTM	334 (1 A/g)	[96]
Hollow structured and flower-like C@MnCo_2_O_4_	HTM	728.4 (1 A/g)	[97]
MnCo_2_O_4_@MnO_2_ core-shell nanowire	HTM	858 (1 A/g)	[98]
MnCo_2_O_4_ cuboidal microcrystals	HTM	600 (0.5 A/g)	[99]
MnCo_2_O_4_ spinel	HTM	671 (5 mV/s)	[100]
1D MnCo_2_O_4_ nanowire	HTM	349.8 (1 A/g)	[101]
PEDOT rod-like@Mn-Co oxide	Deposit	310 (15 mA/cm^2^)	[102]
MnCo_2_O_4_ spinel	Sel-gal	510 (5 mV/s)	[100]
MnCo_2_O_4_ spinel	Sel-gal	405 (5 mA/cm^2^)	[103]
Mesoporous MnCo_2_O_4_ spinel	Solvothermal	346 (1 A/g)	[104]
Spinel MnCo_2_O_4_ nanosheets	Electrodeposition	290 (1 mV/s)	[105]
MnO_2_/MnCo_2_O_4_ nanoparticle	SCS	497 (0.5 A/g)	[94]

**Table 10:** The electrochemical performance of different iron oxide anodes. (Reprinted with permission from [106]. Copyright 2018 Elsevier).

Material	Reversible Capacity (mAh/g)	Current Density (A/g)	Ref.
α-Fe_2_O_3_/Fe_3_O_4_ porous nanosheets	1258 (500 cycles)	1	[106]
3D hierarchical porous α- Fe_2_O_3_ nanosheets	1001 (1000 cycles)	1	[107]
Fe_2_O_3_@chitosan	732 (50 cycles)	0.1	[108]
α-Fe_2_O_3_/SWNT hybrid films	1100 (100 cycles)	0.1	[109]
porous α-Fe_2_O_3_ nanoparticles	841 (100 cycles)	0.5	[110]
graphene@C/Fe_3_O_4_	872 (100 cycles)	0.1	[111]
Fe/Fe_3_O_4_/C nanocomposites	755 (100 cycles)	0.1	[112]
Fe_3_O_4_/C nanosheets	647 (100 cycles)	0.2	[113]

**Table 11:** The electrochemical performance of different iron oxide anodes. (Reprinted with permission from [114]. Copyright 2014 Elsevier).

Sample	S_BET_ (m^2^/g)	Vpore (cm^3^/g)	Vmeso (cm^3^/g)	Vmacro (cm^3^/g)
I	13.1	0.037	0.029	0.008
II	21.3	0.095	0.067	0.028
III	14.1	0.054	0.035	0.019
IV	17.9	0.095	0.052	0.043

## Data Availability

Data are contained within the article.
